# An integrative in-silico analysis discloses a novel molecular subset of colorectal cancer possibly eligible for immune checkpoint immunotherapy

**DOI:** 10.1186/s13062-022-00324-y

**Published:** 2022-05-09

**Authors:** Pasquale Sibilio, Francesca Belardinilli, Valerio Licursi, Paola Paci, Giuseppe Giannini

**Affiliations:** 1 Department of Translational and Precision Medicine, University La Sapienza, 00161 Rome, Italy; 2 Institute for Systems Analysis and Computer Science Antonio Ruberti, National Research Council, 00185 Rome, Italy; 3 Department of Molecular Medicine, University La Sapienza, 00161 Rome, Italy; 4 Department of Biology and Biotechnologies “Charles Darwin”, University La Sapienza, 00185 Rome, Italy; 5 Institute of Molecular Biology and Pathology, National Research Council of Italy, Via degli Apuli, 4, 00185 Rome, Italy; 6 Department of Computer Engineering, Automation and Management, University La Sapienza, 00161 Rome, Italy; 7 Istituto Pasteur-Fondazione Cenci Bolognetti, 00161 Rome, Italy

**Keywords:** Colorectal cancer, Meta-analysis, Immunoinformatics, Multi-omics, Immunotherapy

## Abstract

**Background:**

Historically, the molecular classification of colorectal cancer (CRC) was based on the global genomic status, which identified microsatellite instability in mismatch repair (MMR) deficient CRC, and chromosomal instability in MMR proficient CRC. With the introduction of immune checkpoint inhibitors, the microsatellite and chromosomal instability classification regained momentum as the microsatellite instability condition predicted sensitivity to immune checkpoint inhibitors, possibly due to both high tumor mutation burden (TMB) and high levels of infiltrating lymphocytes. Conversely, proficient MMR CRC are mostly resistant to immunotherapy. To better understand the relationship between the microsatellite and chromosomal instability classification, and eventually discover additional CRC subgroups relevant for therapeutic decisions, we developed a computational pipeline that include molecular integrative analysis of genomic, epigenomic and transcriptomic data.

**Results:**

The first step of the pipeline was based on unsupervised hierarchical clustering analysis of copy number variations (CNVs) versus hypermutation status that identified a first CRC cluster with few CNVs enriched in Hypermutated and microsatellite instability samples, a second CRC cluster with a high number of CNVs mostly including non-HM and microsatellite stable samples, and a third cluster (7.8% of the entire dataset) with low CNVs and low TMB, which shared clinical-pathological features with Hypermutated CRCs and thus defined Hypermutated-like CRCs. The mutational features, DNA methylation profile and base substitution fingerprints of these tumors revealed that Hypermutated-like patients are molecularly distinct from Hypermutated and non-Hypermutated tumors and are likely to develop and progress through different genetic events. Transcriptomic analysis highlighted further differences amongst the three groups and revealed an inflamed tumor microenvironment and modulation Immune Checkpoint Genes in Hypermutated-like CRCs.

**Conclusion:**

Therefore, our work highlights Hypermutated-like tumors as a distinct and previously unidentified CRC subgroup possibly responsive to immune checkpoint inhibitors. If further validated, these findings can lead to expanding the fraction of patients eligible to immunotherapy.

**Supplementary Information:**

The online version contains supplementary material available at 10.1186/s13062-022-00324-y.

## Introduction

Colorectal Cancer (CRC) is a major cause of cancer-related death worldwide, accounting for approximately 8% of all annually diagnosed cancers [1]. Historically, the molecular classification of CRC was based on the global genomic status, which identified three major groups: tumors with microsatellite instability (MSI; ~ 15% of all CRCs), tumors with chromosomal instability (CIN; ~ 85% of all CRCs) and tumors with a CpG island methylator phenotype (CIMP; ~ 20% of all CRCs) [2].

In MSI tumors, defects of the mismatch repair (MMR) pathway are the leading cause of genetic instability. It can be due to inactivating mutations or to epigenetic silencing by promoter hypermethylation of DNA MMR genes [2], a condition frequently associated to high levels of CpG island methylation and referred to as CIMP-High (CIMP-H, ~ 70–85% of MSI CRCs). Defective DNA MMR (dMMR) leads to reduced restoration of replication errors resulting in the introduction of a high rate of mismatches in microsatellites. The consequent changes in microsatellite lengths may be monitored to classify different phenotypes as microsatellite stable (MSS) or unstable (MSI), which can be further subdivided MSI-High (MSI-H) or MSI-Low (MSI-L) [2, 3]. Tumors with MSI-H typically display a high rate of point mutations [4, 5], a state referred to as hypermutation (HM). Besides dMMR, the HM phenotype is also related to somatic or germline mutations of POLE and POLD1 genes encoding DNA polymerase epsilon and delta, respectively [3].

CIN tumors instead bear high frequency of copy number variations (CNVs). In almost all cases they are MSS or MSI-L, usually share low mutation rate, and null or low level of CIMP (non-CIMP or CIMP-L) [2, 6].

Over the years, additional molecular classifications beyond CIN, MSI and CIMP have been proposed with the aim to dissect the heterogeneity of CRC for prognostic and predictive intents [7–11]. In example, the Consensus Molecular Subtypes (CMS) Consortium, analyzing CRC expression profiling data from multiple studies, converged on the definition of four main CMSs [10]. Although CMSs have prognostic and therapeutic implications, they have not been translated into clinical routine, yet.

With the introduction of immune checkpoint inhibitors (ICIs) for the treatment of metastatic CRC (mCRC), MSI/CIN classification regained momentum as the dMMR/MSI-H condition (~ 2–4% of mCRCs) predicted sensitivity to ICIs in clinical trials, possibly due to both high rate of tumor mutational burden (TMB-H) and high levels of infiltrating lymphocytes typically present in these tumors [3, 12, 13]. Conversely, pMMR-MSS/MSI-L a group, as a whole appears resistant to ICIs therapies.

To better understand the differences between MSI/CIN status and eventually discover additional CRC subgroups relevant for therapeutic decisions, we developed a computational pipeline that include molecular integrative analysis of genomic, epigenomic and transcriptomic data of 520 CRC samples downloaded from The Cancer Genome Atlas (TCGA) data portal. The results highlighted a novel non-CIN, non-MSI and CIMP-L CRC subgroup, characterized by KRAS-high/TP53-low mutation rate, distinct mutational signatures and an inflamed tumor microenvironment.

## Materials and methods

### Data collection and processing

We downloaded genomic, transcriptomic and epigenomic data from TCGA-COAD and READ projects stored on TCGA data portal (https://portal.gdc.cancer.gov/), accessed in November 2020. We performed meta-analysis on 520 TCGA-COAD and READ patients of which copy number variations (CNVs), whole exome sequencing (WES), transcriptomic (RNA-seq), DNA methylation and MSI status data were available.

**Fig. 1:**
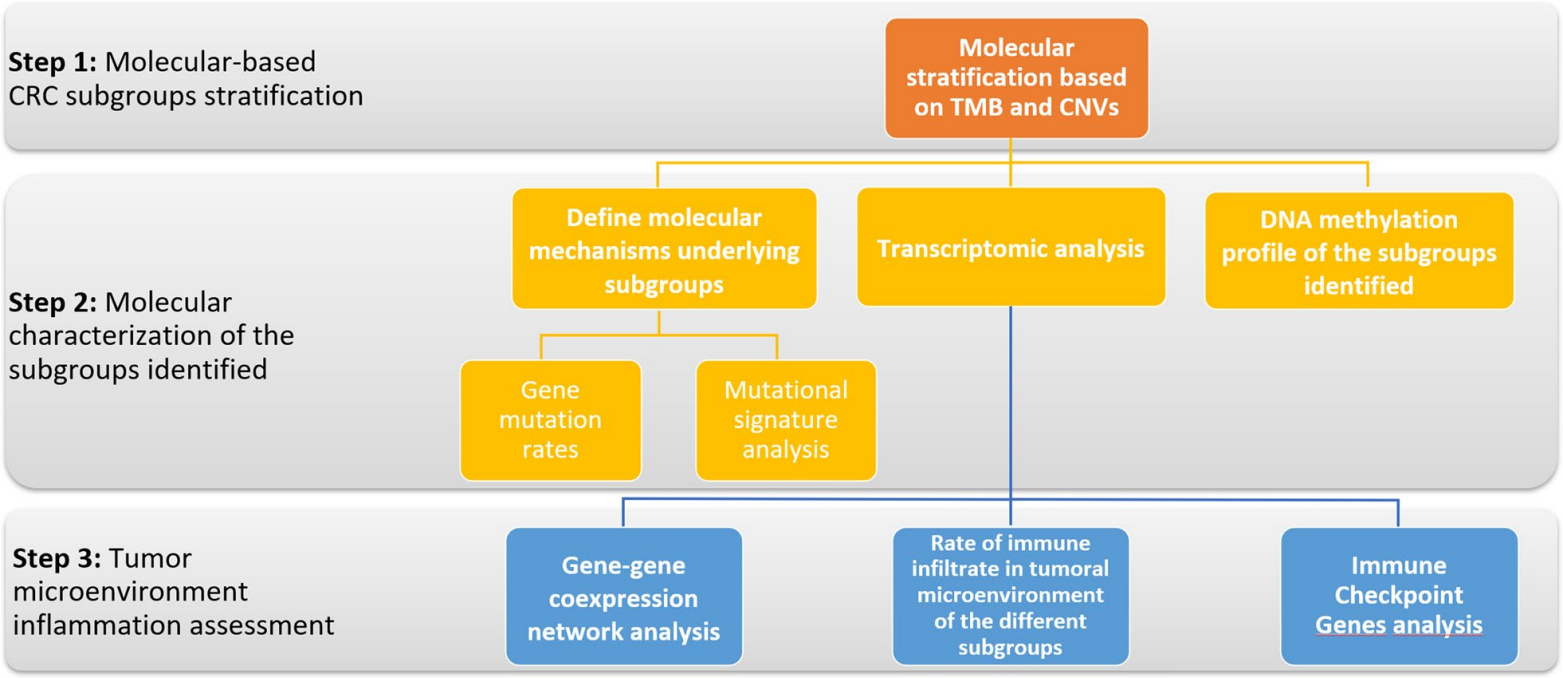
Computational pipeline flowchart

We developed a computational pipeline that includes molecular integrative analysis at genomic, epigenomic and transcriptomic level to better classify patients affected by CRC. The pipeline is subdivided in steps, described in Fig. 1.

#### Step1: molecular-based CRC subgroups stratification

##### Tumor mutational burden (TMB) Analysis

Tumor mutational burden (TMB) was calculated dividing the total number of nonsynonymous mutations of every patient per 30 Megabase, which is the average size of the exome. Numbers of nonsynonymous mutations are derived from MAF files retrieved from TCGA resulting from variant analysis of WES experiments on 520 TCGA-COAD and READ patients. According to [5] patients with a TMB higher and lower than 20 per Megabase were classified as HM or non-HM, respectively.

##### CNV calling and analysis

We performed CNVs calling from segmented mean data employing GISTIC 2.0 which identifies genomic regions that are significantly gained or lost across the 520 TCGA-COAD and READ tumors [14]. For details on how GISTIC calculates focal and broad amplification/deletion in chromosome regions and how the algorithm was set to our data, refer to Additional file 1. The R package *copynumber* [15] was used to visualize the frequency of gain/loss in the chromosome regions among the CRC’s subgroups identified. The association between frequency of CNVs events in the chromosome regions and the CRC’s subgroups identified was evaluated using Fisher’s exact test.

#### Step 2: molecular characterization of the subgroups identified

##### Mutational data analysis

The R package *maftools* [16], which contains functions to perform most used analyses in cancer genomics and to create feature rich customizable visualizations, have been used to analyze MAF files of the 520 TCGA-COAD and READ tumors and to address the mutational signatures. We studied top frequently mutated genes discovered in our cohort plus recurrently mutated genes defined in the COSMIC database [17]. The association between different mutational rates in the genes analyzed and HM, HM-like and non-HM groups was evaluated using Fisher’s exact test. Further, we performed the analysis of the non-silent mutations existing in POLE exonuclease domain from exon 9–14 in the three subgroups.

Other algorithms implemented in the maftools package allowed the extraction of mutational signatures from MAF files and to compare them with the validated signature present in the COSMIC curated database. For details on the evaluation of mutational signatures, refer to Additional file 1.

##### DNA methylation analysis

Data containing β-values from the Illumina Infinium HumanMethylation450 Array were available for 382/520 of the patients enrolled in the study. In pre-processing steps, we filtered out probes containing Single Nucleotide Polymorphisms (SNPs) and designed on X and Y chromosomes. To determine CpG Island Methylator Phenotype (CIMP) status, we first identified the 1000 differentially methylated CpGs between the three groups (ANOVA-like test using limma package) [18]. Afterwards, we computed an unsupervised hierarchical clustering that identified 3 clusters and considered the methylome patterns of the clusters we could assign to cluster 1 to CIMP-Low (CIMP-L), cluster 2 to CIMP-High (CIMP-H) and cluster 3 to non-CIMP (Fig. 3). The hierarchical clustering analysis was performed by using “maximum” as clustering distance and “ward.D2” as clustering method.

#### Step 3: tumor microenvironment inflammation assessment

Weighted gene co-expression network analysis (WGCNA) of the transcriptomic data of 520 TCGA-COAD and TCGA-READ tumors was leveraged by using the R package WGCNA [19, 20]. For details on how the WGCNA algorithm was implemented, refer to Additional file 1.

RNA-seq data of the 520 TCGA-COAD and TCGA-READ patients was leveraged to evaluate the quality and quantity of immune infiltrate in the tumoral environment. Analysis was conducted using *ImSig*, which is a R library that provides functions to study the expression and abundance of immune cells in cancer tissue transcriptomics [21]. This approach incorporates immune/inflammatory cells in 7 major classes (B cells, Interferon, Macrophages, Monocytes, Neutrophils, NK cells, T cells) plus 3 additional signatures (Plasma cells, Proliferation and Translation). A correlation cut-off of 0.8 was used, to remove genes that did not exhibit a strong correlation with the ImSig signatures. Furthermore, to assess the statistical significance of the difference of the mean expression of each immune signature in the multiple comparison of the three groups the Tuckey’s test was used, which is a post-hoc test after ANOVA analysis.

In addition, we studied the expression of 79 Immune Checkpoint Genes (ICGs) curated by [22], in our cohort. A differentially expression analysis was performed using the multiple comparison of the three subgroups using Wald test (Additional file 4: Table S6) and p-value was adjusted according to the Benjamini–Hochberg method. Thresholds for FDR < 0.1 and Log2 Fold Change > 0.4 were used to select significant differentially expressed genes.

## Results

### Classification of the CRC samples according to TMB and CNVs

We subjected 520 CRC tumor samples of COAD/READ projects to TMB analysis. 78/520 (15%) samples were classified as hypermutated (HM: TMB > 20 per 10^6^ bases) with a median value of 44.9 mutations per 10^6^ bases (range: 26–347 per 10^6^ bases), while 442/520 (85%) samples were classified as non-HM with a median value of 3.5 mutations per 10^6^ bases (range: 0.1–24 per 10^6^ bases).

**Fig. 2:**
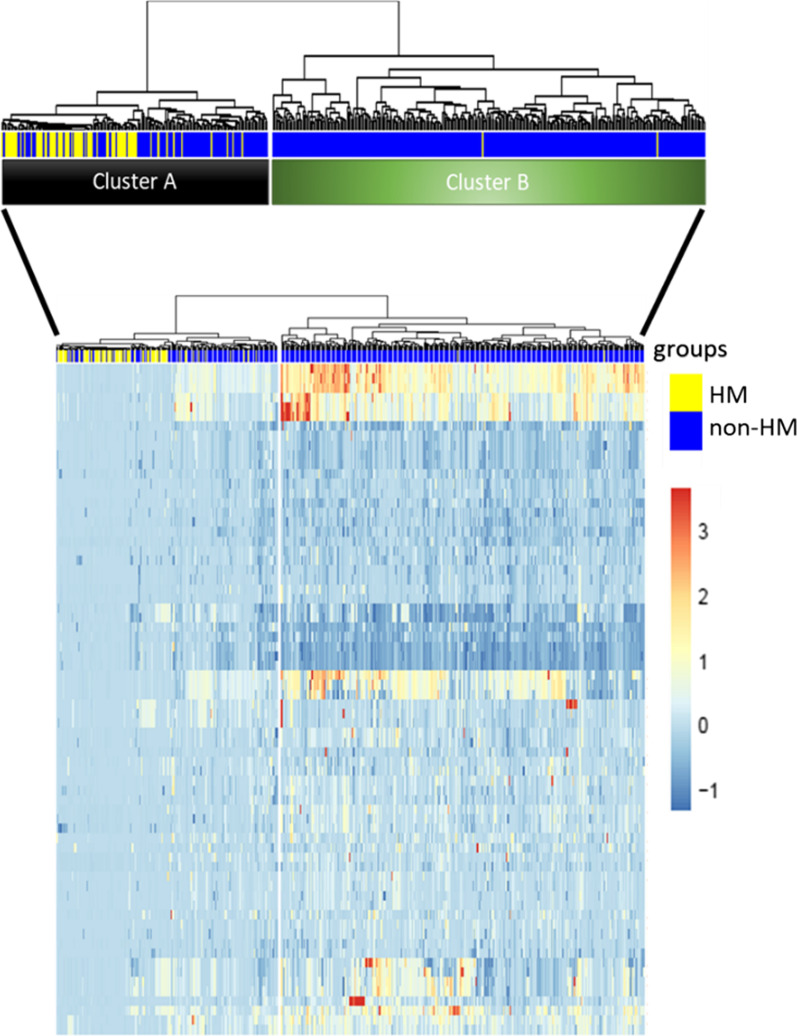
Unsupervised hierarchical clustering analysis based on CNVs data of the 520 CRC patients selected from TCGA-COAD and READ projects. The lines in the heatmap represent significant focal alteration. The columns correspond to the 520 patients. HM and non-HM samples are indicated in yellow and blue colors, respectively. This analysis identified two main clusters: cluster a (ClA) and cluster b (ClB). ClA (117/520; 22.5%) is characterized by a few events of CNVs along the chromosome regions and was enriched in HM samples (n = 76/117; 63.9%). ClB contains samples with a high number of CNVs events and it mostly consists of non-HM samples (n = 401/403; 99%). Among ClA, we identified a sub-group of tumors (called HM-like; n = 41/520; 7.8%) with a similar CNV profile of ClA, also characterized by a low TMB. To the right-hand side of the figure, a scale indicates the color code relative to the log2 segment mean value of CNVs (ranging from − 1 up to 3)

The CNV calling analysis resulted in 29 amplified and 41 deleted focal regions significantly altered through all sets of tumor samples. We then subjected the 520 tumor samples to an unsupervised hierarchical clustering analysis of the CNVs which identified two main clusters (Fig. 2): Cluster A (ClA) characterized by few CNV events and Cluster B (ClB) with a high number of CNVs events. ClA was enriched in HM samples (n = 76/117; 65%; Fig. 2, yellow bars), while ClB mostly contained non-HM samples (n = 401/403; 99.5%; Fig. 2, blue bars). Within ClA, we noted a group of 41 samples with low CNV profile and very low TMB (median value of 3.9 mutations per 10^6^ bases, range: 0.1–23 mutations per 10^6^ bases). Based on their clinical-pathological similarities with HM CRCs (as described below) this subset will be referred to as HM-like (Fig. 2) and accounted for 7.8% of the entire dataset.

**Fig. 3:**
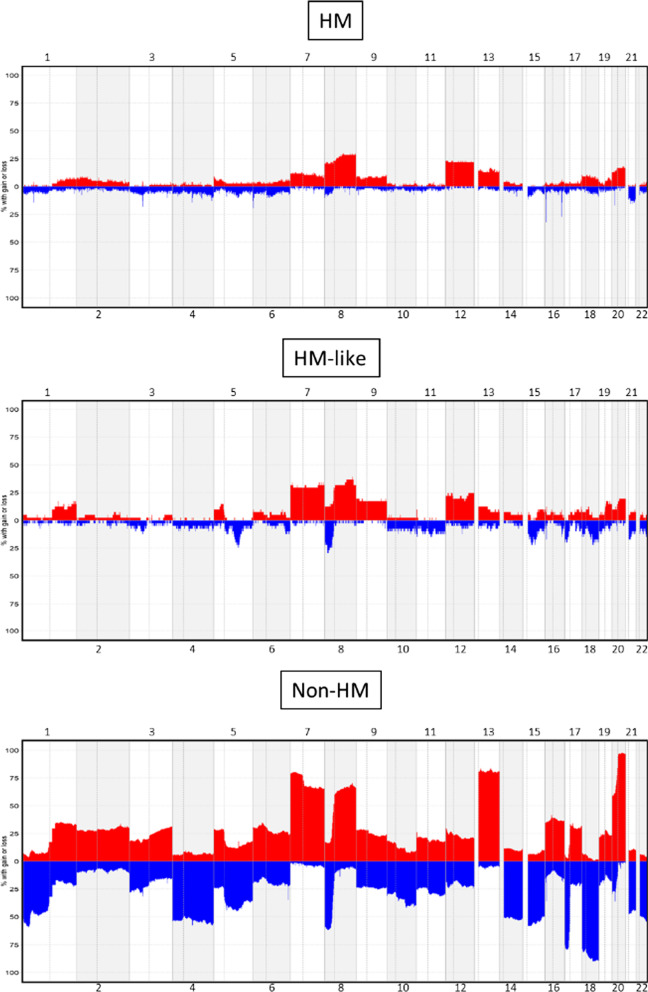
Frequency of CNV events along the genome identified in HM, HM-like and non-HM samples. Frequencies (vertical axis, 0–100%) are plotted as a function of the chromosome location. Copy number gains and losses are highlighted in red and blue, respectively

The profiles of CNVs amount and distribution among chromosomes was clearly distinct between the three subgroups. Overall, the HM-like group was characterized by a CNV profile more similar to the HM group than to the non-HM group (Fig. 3). However, these tumors also showed recurrence of gains (chromosomes 7, 9p and 19q) and losses (chromosome 8p, 10, 11, 15q, 17p and 18) more typical of non-HM samples (Fig. 3, Additional file 1: Tables S1, S2).

As expected, most HM tumors were classified as MSI-H (n = 61/78; 78.2%), while non-HM and HM-like patients were much more frequently MSS stable (n = 377/401; 94.0% and n = 34/41; 82.9%, respectively; Table 1). Consistent with the results of population studies [23], POLE exonuclease domain mutation rate was 2.9% (15/520) in our cohort, and all mutations fell in HM-group (15/78; 19.2%), while non-HM and HM-like patients showed no POLE alteration (Table 1).

**Table 1:** Clinical-Pathological features of HM, HM-like and non-HM groups

		HM (n = 78)	HM-like (n = 41)	Non-HM (n = 401)	*P* value
Stage	I	14 (17.9%)	10 (24.4%)	62 (15.5%)	NS
	II	45 (57.7%)	16 (39.0%)	123 (30.9%)	***
	III	14 (17.9%)	13 (31.7%)	125 (31.2%)	*
	IV	3 (3.8%)	1 (2.4%)	72 (18.0%)	***
	No data	2 (2.6%)	1 (2.4%)	19 (4.7%)	***
Location	Ascending	50 (64.1%)	21 (51.2%)	115 (28.7%)	***
	Transverse	10 (12.8%)	8 (19.5%)	16 (4.0%)	***
	Descending	12 (15.4%)	11 (26.8%)	253 (63.1%)	***
	No data	6 (7.7%)	1 (2.4%)	17 (4.2%)	
Mutational Burden	Median of mutations/Megabase	44.9	3.9	3.5	
MSI-status	MSI-H	61 (78.2%)	6 (14.6%)	3 (0.7%)	***
	MSS/MSI-L	11 (14.1%)	34 (82.9%)	377 (94.0%)	***
	Indeterminate	6 (7.7%)	1 (2.4%)	21 (5.2%)	–
Pol-ε exonuclease domain mutation		15 (19.2%)	0	0	

Overall, this analysis suggests that non-HM and HM subsets largely comprise CRCs associated with typical CIN and MSI/hypermutated phenotypes, respectively, while HM-like tumors appear as a distinct entity, with rather low CNVs and mutation rates.

### Clinical-pathological features and gene mutation rates in HM, HM-like and non-HM samples

Clinical-pathological features of HM, HM-like and non-HM samples are reported in Table 1. No significant associations were found with age or gender. As expected, HM patients were significantly enriched in early stages and in ascending colon localization compared to non-HM patients, which were more associated with stage 4 and in descending colon localization [11, 24]. Intriguingly, HM-like patients shared with HM subset a similar enrichment in early stages, with only 2.4% (1/41) and 3.8% (3/78) of the patients with HM-like and HM profiles in stage 4, against a rate of 18.0% (72/401) for non-HM patients (*P* < 0.0001, Fisher’s exact test). Moreover, HM-like tumors were more frequently associated with ascending colon location (21/41; 51.2%) similar to HM (50/78; 64.1%), in contrast to non-HM tumors which were associated with descending colon location (253/401; 63.1%) (*P* < 0.0001, Fisher’s exact test).

To further compare the overall molecular features of HM-like versus HM and non-HM subsets we examined SNV data. As expected from the literature and according to their CIN profile [25] non-HM tumors had higher mutation rate in APC (84%), TP53 (69%) and KRAS (41%) compared to HM tumors (Table 2). In contrast, HM tumors had high mutation rates in genes of the WNT signaling, TGF-β, PI3K-AKT and MAPK/ERK pathways as well as in ATM, KMT2D and LRP1D [26]. Interestingly, HM-like tumors had the highest frequency in KRAS (59%) and SOX9 (27%) gene mutations compared to the other groups. Also, they showed the lowest TP53 mutation rate (15%) and a rate of APC mutations similar to HM samples and significantly lower than non-HM samples (Table 2).

**Table 2:** Mutational rate of most frequently altered genes in CRC in HM, HM-like and non-HM group

Genes	Pathway	HM (%)	HM-like (%)	Non-HM (%)	*P* value
APC	WNT signaling	49	59	84	***
AMER1	WNT signaling	27	15	9	***
CTNNB1	WNT signaling	24	12	3	***
TCF7L2	WNT signaling	24	0	7	***
FBXW7	WNT signaling	40	32	11	***
ARID1A	WNT signaling	45	5	6	***
SOX9	WNT signaling	15	27	11	*
TGFBR2	TGF-β signaling	12	7	1	NS
ACVR2A	TGF-β signaling	37	15	1	**
SMAD4	TGF-β signaling	15	17	12	NS
PIK3CA	PIK3 signaling	40	41	21	***
PTEN	PIK3 signaling	22	10	3	**
FAT4	Hippo signaling pathway	76	24	15	***
ERBB2	MAPK signaling	15	5	2	***
ERBB3	MAPK signaling	22	5	2	***
KRAS	MAPK signaling	26	59	41	***
NRAS	MAPK signaling	4	7	7	NS
BRAF	MAPK signaling	62	12	3	***
ATM	DNA damage response	50	10	7	***
TP53	DNA damage response	29	15	69	***
LRP1B	Membrane trafficking	53	5	13	***
KMT2D	Histone methyl transferase	64	15	3	***

The pattern of mutational targets and rates support the hypothesis that HM-like tumors may represent a distinct subgroup of CRCs, which may develop and progress through a different sequence of genetic events compared to the well-known MSI/hypermutated and MSS/CIN subsets, while sharing prevalence of early stages and ascending colon localization with the HM subset.

### Fingerprints of base substitutions in HM, HM-like and non-HM groups reveals unique mutational signature for each group

To further question whether HM-like CRCs are distinct from MSI/hypermutated and MSS/CIN subsets, we searched for the emergence of specific mutational signatures in the three subgroups. Indeed, different mutational processes generate unique combinations of base changes, termed “Mutational Signatures” which can be used as a readout of the biological history of a cancer [27]. To define the mutational signatures associated with HM, HM-like and non-HM groups we performed a classification of base substitutions to include the 3′ and 5′ flanking bases at the mutated site [16]. Thus, we extracted 3 mutational signatures from each group and compared them to COSMIC Single Base Substitution (SBS) Signatures database, a catalog of known mutational signatures identified from > 12,000 samples derived from 40 types of human cancer in which additional information for each signature were also provided.

The top three signatures extracted from the HM group were the most similar to COSMIC SBS6, SBS10b and SBS44 signatures (Table 3) and that is consistent with the “hypermutated” phenotype defining the HM group, since SBS10b signature is associated with POLE mutations, which outbreaks in a high mutational rate, and COSMIC SBS6 and SBS44 are typically associated with dMMR.

**Table 3:** Records of the cosine similarity between the three mutational signatures extracted from each group from the MAF files and the three most similar COSMIC mutational signatures

	SBS best match	Aetiology	Cosine similarity
HM			
Signature 1	SBS44	Defective DNA mismatch repair	0.81
Signature 2	SBS10b	Polymerase epsilon exonuclease domain mutations	0.78
Signature 3	SBS6	Defective DNA mismatch repair	0.90
HM-like			
Signature 1	SBS1	Spontaneous or enzymatic deamination of 5-methylcytosine	0.94
Signature 2	SBS30	Deficiency in base excision repair due to inactivating mutations in NTHL1	0.83
Signature 3	SBS6	Defective DNA mismatch repair	0.93
Non-HM			
Signature 1	SBS1	Spontaneous or enzymatic deamination of 5-methylcytosine	0.96
Signature 2	SBS40	Unknown	0.89
Signature 3	SBS6	Defective DNA mismatch repair	0.77

In the table are reported the best matches between the three mutational signatures extracted from the three groups and the COSMIC SBS Signatures database

The three signatures extracted from non-HM samples had the highest similarity with COSMIC SBS1, SBS6 and SBS40 signatures (Table 3). SBS1, which was also noted in the other subgroups, is related to the spontaneous or enzymatic deamination of 5-methylcytosine to thymine and is widespread in many tumors. SBS40 signature is not clearly associated with a specific etiology, but like SBS1 it is widespread in most cancers and shows some relationships with the age of patients [28].

The three signatures extracted from HM-like samples showed high similarities with SBS1, SBS6 and SBS30. In particular, similarity to SBS30 represents a feature unique to HM-like samples (Table 3). This signature has been recently associated with deficiency in the base excision repair and in particular with inactivation of the NTHL1 gene [29]. Despite some similarities in the mutational signatures were shared by two or even all three subgroups (i.e. SBS1, SBS6 and SBS15), this analysis further evidenced distinct mutational profiles between the HM, HM-like and non-HM subgroups.

### Different CpG methylation patterns occur in the three CRC subgroups

**Fig. 4:**
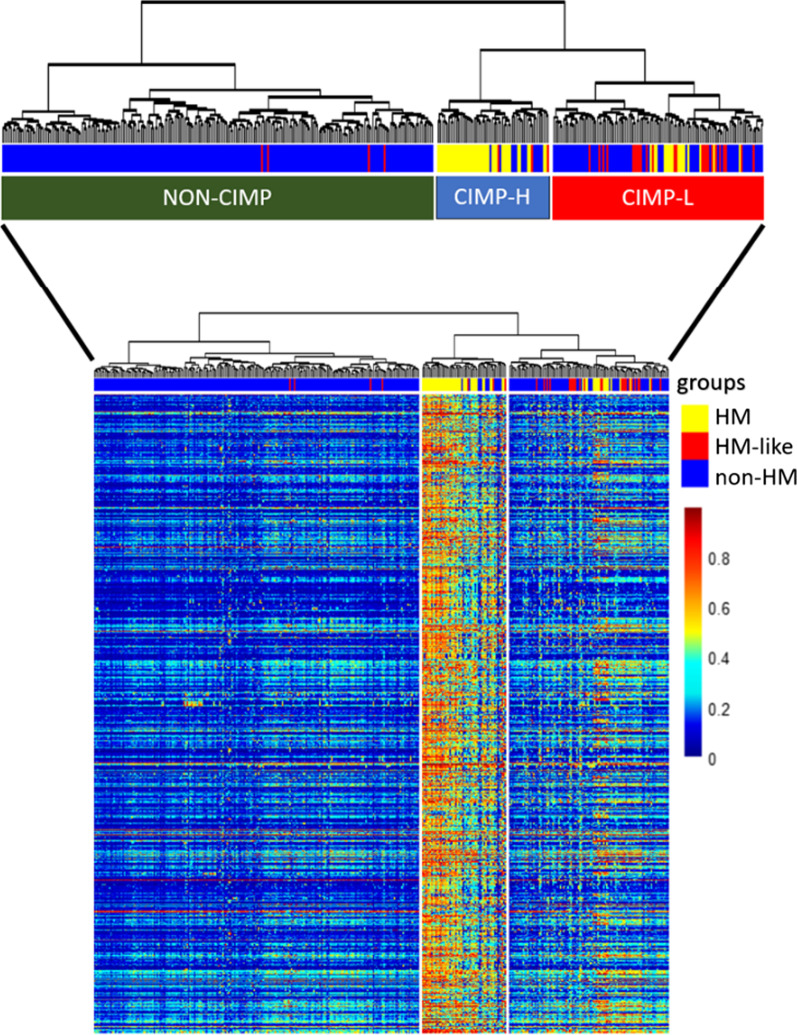
Unsupervised hierarchical clustering analysis based on CpGs methylation data of the 382 patients selected from TCGA-COAD and READ projects. The lines on the heatmap represent the 1000 most differentially methylated CpGs probes between HM, HM-like and non-HM groups. The columns correspond to the 382 patients. Inside the cells of the heatmap are reported the β-values which represent the methylation rate of the probes. The HM patients are reported in yellow; the HM-like patients in red while the non-HM patients in blue. The hierarchical clustering dendrogram supported three distinct tumour groups: CIMP-H (n = 57) defined by an high rate of CpGs probes methylated; CIMP-L (n = 107) with low rate of CpGs probes methylated and non-CIMP (n = 218) characterized by the absence of CpGs probes methylated

Next, we performed an unsupervised hierarchical clustering analysis for 382 of the TCGA-COAD/READ samples for which CpGs methylation data were available. The hierarchical clustering dendrogram defined three distinct tumor groups: CIMP-H (n = 57/382; 14.9%) with a high rate of CpGs probes methylated; CIMP-L (n = 107/382; 28.0%) with low rate of CpGs probes methylated and non-CIMP (n = 218/382; 57.1%) characterized by the absence of CpGs methylated probes (Fig. 4 and Additional file 1: Fig. S1). As expected, most of the HM patients belong to the CIMP-H cluster (41/57; 71.9%) and most non-HM tumors belong to the non-CIMP cluster (214/294; 72.8%), while a small number of HM and non-HM tumors clustered in the CIMP-L group. Interestingly, we revealed that the HM-like samples are mainly associated with CIMP-L phenotype (24/31; 77.4%) (Fig. 4; Additional file 1: Fig. S2). These results highlighted a different methylome pattern of HM-like tumors compared HM and non-HM.

### WGCNA analysis supports HM, HM-like and non-HM tumors as three distinct CRC subgroups

**Fig. 5:**
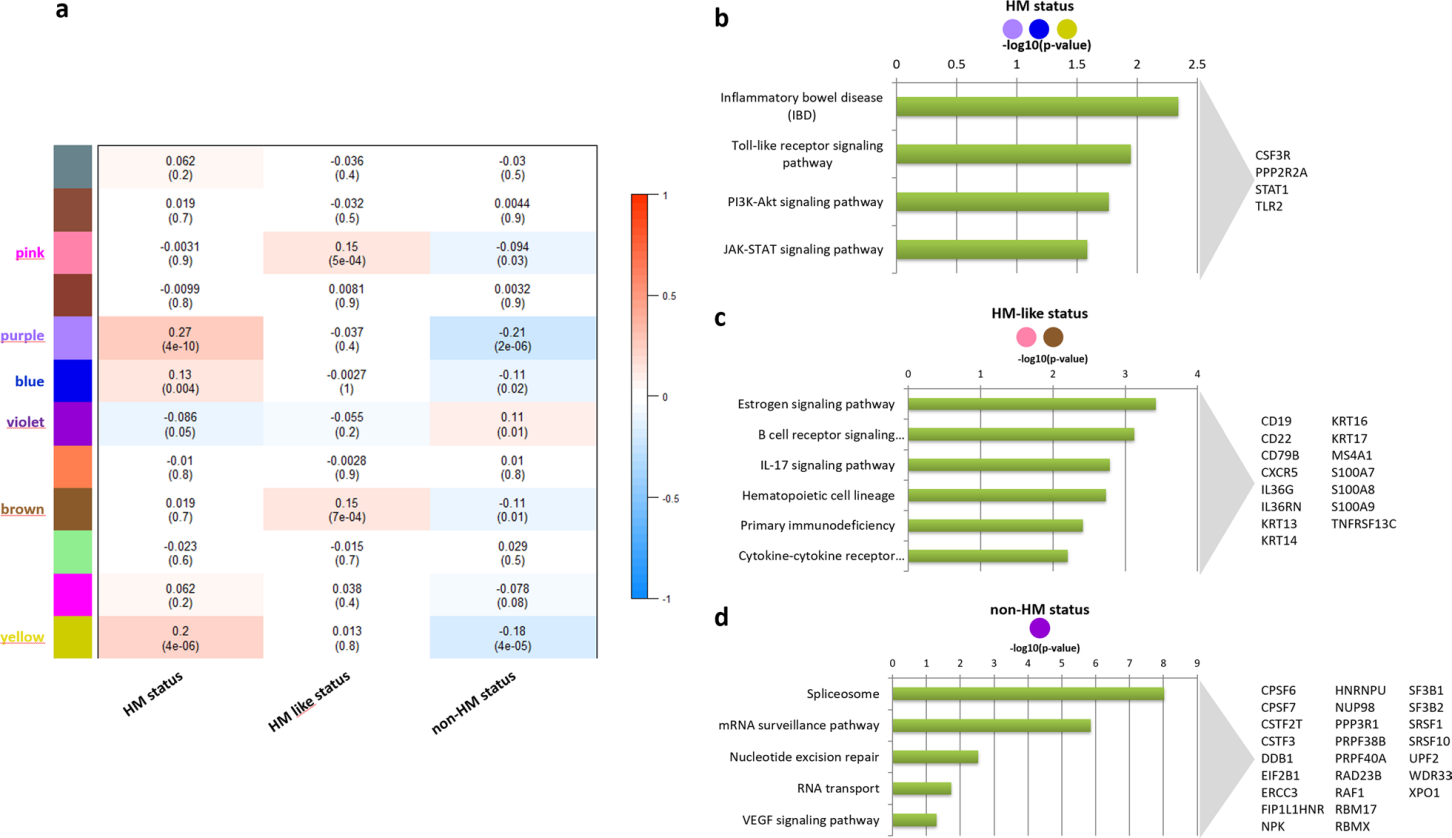
WGCNA analysis. **a** Heatmap of module-trait associations. In the heatmap, each row corresponds to a module eigengene and each column to a trait. Each cell contains the corresponding correlation and *P* value. The table is color-coded by correlation according to the color legend. The traits along the columns have been numerically encoded as follows: HM status (no = 1, yes = 2); HM like status (no = 1, yes = 2); non-HM status (no = 1, yes = 2). The colour labels of modules with at least one statistically significant correlation were highlighted. **b**, **c** KEGG pathways. Results of KEGG pathways enrichment analysis for the most representative genes (module membership > 0.9) falling within the modules statistically significant correlated with the HM status (**b**), HM like status (**c**), and non-HM status (**d**). The names of genes annotated for the enriched KEGG pathways were reported

We performed the WGCNA network-based methodology on the transcriptomic data of 520 TCGA-COAD/READ patients. This analysis revealed 12 highly correlated modules within the gene correlation network, which encompassed genes that were more correlated among each other than with other nodes in the network. For each module, through the WGCNA analysis, we computed the module eigengene defined as the first principal component of that module. By considering as external clinical traits the HM, HM-like, and non-HM status, we then computed the Pearson correlation coefficient between the module eigengene of each module and these external traits (Fig. 5a). We found (1) three modules with statistically significant positive correlations with the HM status, meaning that genes belonging to these three modules were highly expressed in HM patients; (2) two modules with statistically significant positive correlations with the HM-like status, whose genes were highly expressed in HM-like patients; (3) one module with a statistically significant positive correlation with the non-HM status, whose genes were highly expressed in non-HM patients. All these modules did not overlap among the patient status (i.e., HM, HM-like, non-HM), suggesting that these three classes of CRC patients are different also with respect to the gene expression data. In order to identify specific gene signatures of the three subgroups, for each gene we computed the module membership (MM) as the correlation between its gene expression profile and the module eigengene and sorted genes within their own modules according to the MM (Additional file 2: Table S3). Yet, we considered as representative genes of a given module the ones whose MM is greater than 0.7.

Then, for each patient status, we grouped together the representative genes of the modules with the highest correlation and performed a functional enrichment analysis. Via this process, we associated putative biomarkers and functional pathways to each status (i.e., HM, HM-like, non-HM). Also, this analysis confirmed relevant differences among the three subgroups. In detail, the HM status was characterized by high expression of genes mainly involved in the inflammatory bowel disease, Toll-like receptor signaling pathway, PI3K-Akt signaling pathway and JAK-STAT signaling pathways (Fig. 5b, Additional file 3: Table S4). The HM-like status was characterized by high expression of genes mainly involved in estrogen signaling and pathways related to the immune/inflammatory response (Fig. 5c, Additional file 3: Table S4). The non-HM status was characterized by high expression of genes mainly involved in RNA processing, DNA repair and VEGF signaling pathway (Fig. 5d, Additional file 3: Table S4).

### Rate of immune infiltrate in tumoral microenvironment of the three CRC subgroups

**Fig. 6:**
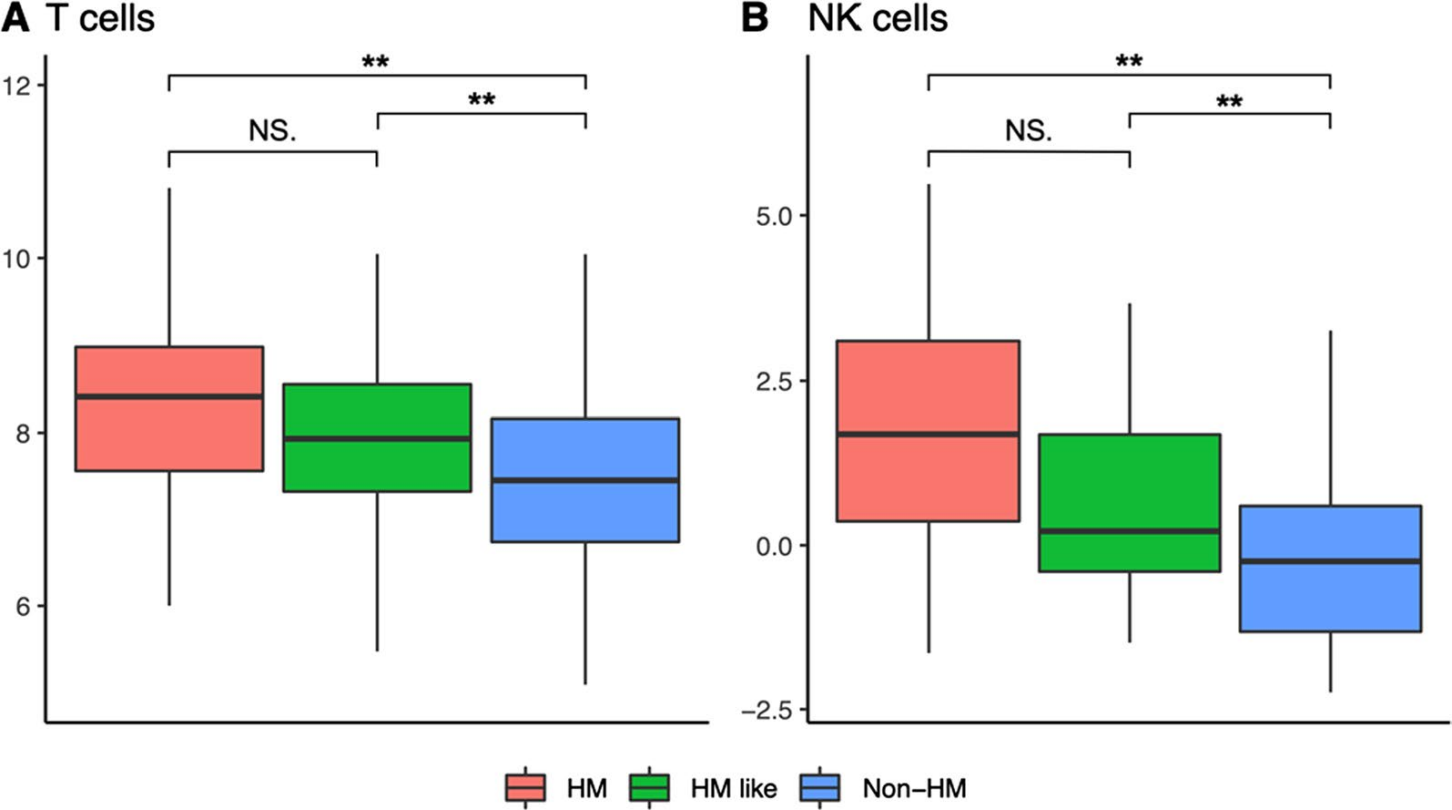
Results of immune signatures analysis performed by ImSig. The boxplots (A and B) show the gene expression of T and NK signature genes (estimated relative abundance) across the HM, non-HM and HM-like groups. Statistical analysis of data was performed using analysis of variance (ANOVA) followed by multiple comparison Tukey’s test. **P < .01, *P < .05

dMMR CRC, largely clustering in the HM subgroups, are typically associated with immune infiltration and good response to ICB therapy [3]. WCGNA analysis indicated activation of inflammatory/immune response genes in HM and HM-like tumors. Therefore, we set out to determine the rate of immune/inflammatory infiltration more specifically in the three subsets by a computational analysis of tumor transcriptomic data, using the R package *ImSig* [21]. By this mean, 10 signatures describing the relative abundance and statistical analysis of 7 inflammatory/immune cells plus 3 additional signatures were analyzed. Concerning T and NK lymphocytes, as expected, we observed the highest signature representation in the HM group, while non-HM have a significantly lower degree of immune cell infiltration (*P* < 0.01) (Fig. 6 and Additional file 1: Fig. S3). Interestingly, HM-like tumors showed T and NK cell signatures similar to HM samples and significantly different than non-HM group (*P* < 0.01). On the other side, HM-like tumors had proliferation, macrophage and interferon signatures more similar to non-HM than HM tumors. However, some genes belonging to interferon signatures and involved in inflammatory/immune responses shared a similar expression between HM-like and HM tumors, while being different from non-HM tumors (Additional file 1: Table S5).

**Fig. 7:**
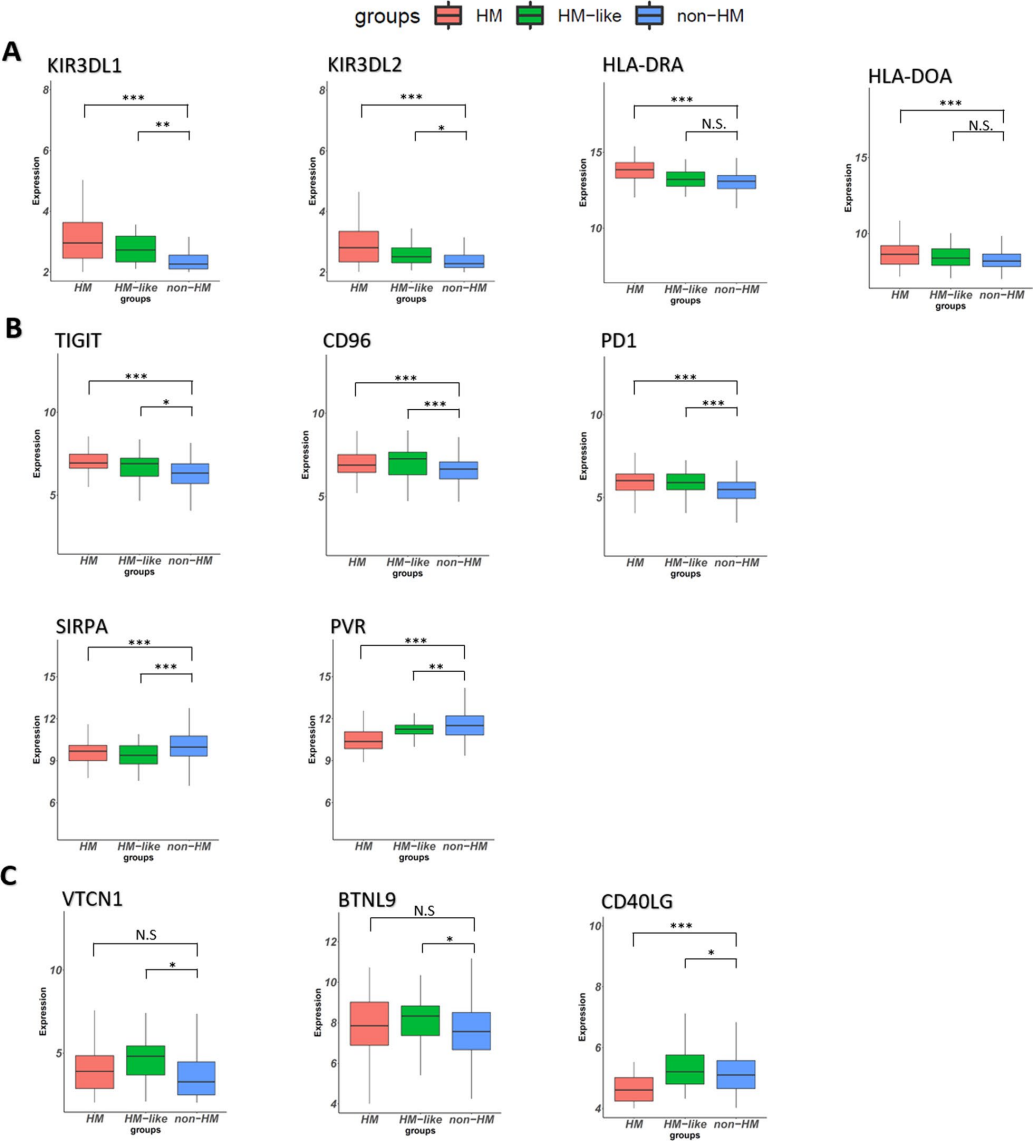
Example of ICGs differentially expressed in HM, non-HM and HM-like groups. The Box Plots show **A** ICGs more expressed in HM group versus non-HM, which may (KIR genes) or may not (HLA genes) be significantly more expressed in HM-like vs non-HM tumors; **B** gene sharing a similar trend of expression between HM and HM-like; **C** gene specifically more expressed in HM-like group (VTCN1 and BTNL9) or with an opposite trend of expression in HM versus HM-like (CD40LG). The analysis was performed using the multiple comparison of the three subgroups using Wald test and *P* value was adjusted according to the Benjamini–Hochberg method. Thresholds for FDR < 0.1 and Log2 Fold Change > 0.4 were used to select significant differentially expressed genes

Next, we performed a differential expression analysis of the 79 ICGs described by [22] expressed in the series (Additional file 4: Table S6). The comparison between HM and non-HM samples revealed that 46 ICGs were differentially expressed and as expected, most of these (29/46; 63.0%) were more expressed in HM group than non-HM. These included KIR and HLA genes, possibly suggestive of NK and antigen presenting cells infiltration, as well as multiple genes directly involved in immune checkpoint regulation, including the well-known PD-L1, PD1, CTLA4, LAG3, TIM3 and TIGIT (Additional file 4: Table S6 and Fig. 7). Interestingly, 17 genes were significantly less expressed in HM compared to non-HM samples (Additional file 4: Table S6). HM-like tumors profoundly differed from HM and non-HM samples. They showed 13 ICGs significantly more expressed compared to non-HM tumors. KIR genes, TIGIT, PD1 and CTLA4 show a similar trend compared to HM samples. Differences in the expression of HLA genes did not reach statistical significance, while CD96 appears even more differentially expressed in this subgroup than in HM tumors, comparing with non-HM subset. Remarkably, we noticed that 4 genes whose role in immune checkpoint regulation is emerging (VTCN1, BTNL9, BTLA and CD28) are specifically more expressed in HM-like group compared to non-HM samples (Additional file 4: Table S6 and Fig. 7). Also, in this comparison we found repressed genes (i.e. SIRPA, BTN2A1 and PVR), some of which followed the same trend of HM tumors, while others where rather specific for this subset (i.e. CD70, CD40). Conversely, IDO1, TDO2 and CD40LG expression trend was completely opposite in HM versus HM-like subgroups.

## Discussion

The comprehension of the biological processes underlying cancer evolution and the molecular stratification of tumors is extremely relevant for prognostic and therapeutic purposes. To this end, the broad inter/intra-tumor molecular heterogeneity of CRC has been widely explored by NGS-based genomic and transcriptomic profiling. Reflecting the different biology of CRCs and their major molecular differences, several molecular classifications with prognostic and predictive value have been proposed [7–11].

One major distinction remains between CIN, MSI and CIMP-H CRCs, with CIN and MSI being mostly mutually exclusive, while CIMP-H largely but incompletely overlaps with MSI CRCs. Despite extensive investigations, this classification remained of poor clinical value until the introduction of ICIs therapies for the treatment of mCRC. In this frame, MSI/CIN classification gained clinical relevance since dMMR/MSI-H CRCs are often responsive to ICIs, probably due to both high TMB (hypermutation status) and high levels of TILs, which are typical hallmarks of these tumors [12, 13]. Indeed, the high mutation load of these tumors might lead to generation of a high number of immunogenic neoantigens [23], which in turn could facilitate immune responses against cancer cells. Conversely, CIN CRCs usually bear low TMB and are mostly resistant to ICIs.

By performing hierarchical clustering analysis of CNVs versus hypermutation status exploiting TCGA CRC datasets, we identified a third cluster of CRCs (7.8%) characterized by low CNVs and low TMB, distinct from the HM and non-HM subsets, which largely match the MSI and CIN groups, respectively. Since this new cluster shares clinical-pathological features with HM CRCs, it was named HM-like subset.

Interestingly, HM-like tumors also showed a distinct mutational profile compared with HM and non-HM tumors, for which we highlighted profiles essentially in line with the literature [11, 30]. In example, the rate of APC mutations in HM-like tumors was similar to HM samples and significantly lower than non-HM samples, while mutations in alternative targets of the WNT and TGF-beta pathways were much lower than those occurring in HM samples, suggesting that this tumor subset is probably less dependent from WNT activation than the other groups. Most importantly, HM-like tumors are characterized by the highest rate of KRAS mutation, a feature that has been previously noted in CIMP-L CRCs [31]. This is a CRC subset with a yet poorly defined clinical relevance, often grouped with the non-CIMP tumors in various studies [32] and sharing the majority of methylation targets with CIMP-H tumors [33]. By methylation analysis, we found that HM-like tumors had mainly a CIMP-L phenotype, at variance with HM and non-HM tumors, which were mostly associated with CIMP-H and non-CIMP phenotype, respectively [11]. Therefore, our data confirm a particularly high recurrence of KRAS mutations in a specific subset of CRCs, associated with CIMP-L phenotype. While the molecular background for this association is not understood yet, recent studies seem to indicate that the strong association between BRAF mutations and CIMP-H phenotype might be due to the need to suppress a senescence-inducing gene expression program promoted by mutant BRAF [34]. Oncogenic RAS molecules are also known to activate senescence in untransformed cells [35, 36]. It is tempting to speculate that also the relevant overlap between KRAS mutation and the CIMP-L phenotype in the HM-like subgroup could be related to the repression of a similar senescence-inducing gene expression program. Further efforts will be required to formally prove this hypothesis.

HM-like CRCs also showed the highest frequency of SOX9 gene mutations and the lowest rate of TP53 mutations. This association has been previously recognized, but its functional significance remains ununderstood [37].

Overall, the genetic marks of HM-like supports the hypothesis that may represent a distinct subgroup of CRCs, which may arise and progress through a different sequence of genetic events compared to the well-known MSI/hypermutated and MSS/CIN subsets. This is further supported by the analysis of mutational signatures, which indicate their unique similarity to the SBS30 pattern. This was recently associated with deficiency in the base excision repair and with inactivation of the *NTHL1* gene [29]. Biallelic *NTHL1* mutations are responsible for the *NTHL1-*tumor syndrome, a cancer-predisposing disease characterized by the occurrence of adenomatous polyposis and cancer at different sites, in addition to CRC [38]. This specific genetic fingerprint indicates that also the pathogenic mechanisms and the etiology underlying HM-like CRCs might be distinct from those leading to HM and non-HM CRCs. So far, we were unable to pull out genomic or transcriptomic alterations in the *NTHL1* gene specifically occurring in the HM-like group, suggesting that functional inactivation of its pathway perhaps associated to the specific CIMP-L pattern might be involved in this respect. Additional studies should be implemented to highlight possible genetic/epigenetic hits or alternative/parallel pathways to *NTHL1* inactivation, which might end up in eliciting the same molecular fingerprints.

The existence of a small group of pMMR/MSS CRCs (~ 10%) responsive to ICIs therapies has been inferred in several clinical studies [39–41]. Pagès and collaborators observed a high immunoscore in 21% of MSS compared to 45% of MSI [42]. Similar findings were reported by Kikuchi et al. which identified a subset of MSI‐L/MSS CRCs within the TCGA COAD/READ dataset showing upregulation of the IFN‐γ and CD8 T effector gene signatures [43]. They also confirmed the presence of a small fraction (~ 12%) of pMMR CRCs positive for PD‐L1 and p‐STAT1 showing increasing grades of infiltrating CD4(+) or CD8(+) TILs on a population of 219 CRC samples.

Our work raised the question whether the HM-like group identifies the same CRC subset. Indeed, not only WCGNA analysis of the transcriptome evidenced relevant differences among the three groups, but also indicated that the HM-like tumors bore high expression of genes associated with immune/inflammatory response. To better investigate this latter aspect, we defined the immune/inflammatory infiltration signature in the three subsets, according to [21]. Intriguingly, we confirmed that HM-like tumors showed T and NK cells signatures similar to HM samples which, as widely known, are inflamed tumors well responsive to ICI therapies. In contrast, proliferation, macrophage and interferon signatures in HM-like tumors were on average more similar to the non-HM than to HM group.

Data on the differential expressions of the ICGs curated by [22] further confirmed the outstanding differences among the three groups. Coherently with the immune infiltration analysis, HM samples showed a high expression of multiple ICGs, confirming the presence of an immune/inflammatory infiltrate (KIR and HLA genes) and differential expression of immune response modulators, including those targeted by established ICI therapies. KIR genes and ICGs (i.e., PD1, CTLA4, CD96 and TIGIT) for which specific targeting therapies have been introduced in the clinical practice [3, 44] also showed a higher expression in HM-like tumors compared to non-HM samples, supporting their immune/inflammatory infiltration. Moreover, our analysis highlighted ICGs exclusively expressed in HM-like, e.g. VTCN1, PCDCD1, CD96, BTNL9 and BTLA, encoding for important immune regulators of both stimulatory and inhibitory pathways, some of which are emerging as new promising targets for immunotherapy [45, 46]. While these data confirm the presence of an immune/inflammatory infiltrate in HM-like tumors showing modulation of established and potentially new immune checkpoint targets to consider for ICI therapies, remarkable differences emerged between HM-like and HM group. Among them, the relatively lower expression of HLA genes in HM-like samples is in line with the poor macrophage signature observed in this subgroup compared to HM samples. The significance of a potentially lower infiltration by antigen presenting/dendritic cells and the relevant differences in the pattern of immunomodulating molecules expressed in HM and HM-like tumors cannot be easily interpreted at the time being and definitely requires further investigations. These differences, however, do not contrast with our hypothesis that HM-like CRCs might be responsive to ICI. Of relevance, the strong negative regulation of IDO and TDO2 in HM-like compared to both HM and non-HM tumors suggest that the formers are possibly characterized by a less immunosuppressive microenvironment caused by the release of tryptophan metabolites. Perhaps this condition might also be related to the more frequent association of HM-like tumors with early stages CRC and may eventually make them more prone to immune reactivation.

Unfortunately, a major limitation of this study is represented by the lack of a univocal specific molecular biomarker/s facilitating the identification of HM-like CRC, in clinical settings. To this end, the possibility to use CIMP-L phenotype needs to be explored.

## Conclusions

Our work indicates the existence of a previously unidentified CRC subgroup with distinctive features and possibly responsive to current or to be defined ICIs. If validated by experimental work, these findings can lead to expanding the fraction of patients eligible to ICIs treatment.

## Supplementary Information

**Additional file 1.** **Supplementary methods; Supplementary Table 1, 2, 5 and Supplementary figures 1,2 and 3**. Descriptions of the supplementary tables and figures are embedded in the file.

**Additional file 2.** **Supplementary Table 3.** This table reported the results of WGCNA analysis.

**Additional file 3.** **Supplementary Table 4.** This table reported the functional enrichment results of the genes falling in the WGCNA - detected modules.

**Additional file 4.** **Supplementary Table 6.** ICGs differentially expressed in the comparison HM vs non-HM and HM-like vs non-HM groups.

## Data Availability

All data generated or analyzed in this study are included in this article (and its supplementary information files), and are available from the corresponding author on request.
