# Supplementary material for: An integrative in-silico analysis discloses a novel molecular subset of colorectal cancer possibly eligible for immune checkpoint immunotherapy
Source: Biol Direct. 2022 May 9;17:10. doi: 10.1186/s13062-022-00324-y (PMC9082922; doi:10.1186/s13062-022-00324-y)
Supplement: Supplementary file 1 — Additional file 1. Supplementary methods; Supplementary Table 1, 2, 5 and Supplementary figures 1,2 and 3. Descriptions of the supplementary tables and figures are embedded in the file. [file 13062_2022_324_MOESM1_ESM.docx]

**Supplementary methods**

***CNV calling and analysis details***

GISTIC 2.0 takes segmented copy number ratios as input, separates arm-level events from focal events and then performs two tests: (i) identifies significantly amplified/deleted chromosome arms; and (ii) identifies regions that are significantly focally amplified or deleted.

For the focal analysis, the significance levels (Q values) were calculated by comparing the observed gains/losses at each locus to those obtained by randomly permuting the events along the genome to reflect the null hypothesis that they were all 'passengers' and could have occurred anywhere. The locus-specific significance levels were then corrected for multiple hypothesis testing. The arm-level significance is calculated by comparing the frequency of gains/losses of each arm to the expected rate given its size. The method outputs genomic views of significantly amplified and deleted regions, as well as a table of genes with gain or loss scores.

Gistic’s parameters used in this study are the following:

*-b “path file* ; *-seg “filename”*; *-refgene refgenefiles/hg19.UCSC.add_miR.140312.refgene.mat* ; *-mk genome.info.6.0_hg19.na31_minus_frequent_nan_probes_sorted_2.1.txt; -ta 0.1; -td 0.1; -broad 1; -brlen 0.7; -twoside 1; -maxseg 2000; -conf 0.95; -genegistic 1; -armpeel 1; -savegene 1*

The R package *copynumber* [21] was used to identify chromosomal regions with gains or loss in our groups of patients. The association between different frequency of CNVs events in the chromosomal regions and HM, HM-like and non-HM groups was evaluated using Fisher’s exact test.

***Mutational signatures extraction and comparison to COSMIC SBS database***

From MAF files a mutation matrix is formed using the six substitution subtypes: C>A, C>G, C>T, T>A, T>C, and T>G and adding information on the bases immediately 5’ and 3’ to each mutated base generating 96 possible trinucleotide types. Then, the signatures are extracted for each group using non-negative matrix factorization to decompose the matrix into 3 signatures. This number of signatures extracted depended on NMF analysis and measures of the goodness of fit in terms of Cophenetic correlation. Then extracted signatures can be compared to known mutational signatures from COSMIC SBS database [9], and cosine similarity was calculated to identify similarities.

**WGCNA analysis**

Weighted gene co-expression network analysis (WGCNA) is one of the most employed algorithm to construct gene co-expression networks across gene expression data, exploring the association between gene networks and phenotypic/clinical traits of interest [25], [26]. WGCNA can identify modules of highly interconnected, or co-expressed, genes within the gene co-expression network by grouping together the most similar nodes. The similarity measure between two nodes is expressed in terms of their direct connection strength as well as connection strengths “mediated” by shared neighbors. The relationship between modules can be studied by correlating the corresponding module eigengenes (MEs). The ME is defined as the first principal component of a given module and can be considered a representative of the gene expression profiles in that module. For each gene are defined two useful measures: module membership (MM) and gene significance (GS). MM is defined by correlating its gene expression profile with the module eigengene of a given module and can be computed for all input genes. If MM of a given gene with respect to a given module is close to 0, that gene is not part of that module. On the other hand, if MM is close to 1 or -1, the gene is highly positive or negative connected to the genes of that module. Finally, to incorporate external information into the co-expression network, WGCNA makes use of gene significance (GS) measures computed as the correlations between gene expressions and external sample traits. Abstractly speaking, the higher the absolute value of GS of a given gene, the more biologically significant that gene is. To perform the WGCNA analysis on the transcriptomic data of 520 TCGA-COAD and TCGA-READ patients, we used the R package WGCNA [26].

**Supplementary tables**

Supplementary table 1. Amplification frequency of chromosome arms in HM, HM-like and non-HM tumors.

| Chromosome arm | HM (%) | HM_like | non_HM | P-value |
| --- | --- | --- | --- | --- |
| 1p | 0 | 1 (2,4%) | 23 (5,7%) | NS |
| 1q | 4 (5,1%) | 3 (7,3%) | 81 (20%) | ** |
| 2p | 4 (5,1%) | 2 (4,8%) | 94 (23%) | *** |
| 2q | 2 (2,5%) | 1 (2,4%) | 95 (24%) | *** |
| 3p | 1 (1,2%) | 0 | 55 (14%) | *** |
| 3q | 1 (1,2%) | 0 | 78 (20%) | *** |
| 4p | 0 | 0 | 16 (3,9%) | NS |
| 4q | 1 (1,2%) | 0 | 13 (3,2%) | NS |
| 5p | 5 (6,4%) | 3 (7,3%) | 81 (20%) | ** |
| 5q | 3 (3,8%) | 0 | 50 (12%) | ** |
| 6p | 3 (3,8%) | 2 (4,8%) | 98 (24%) | *** |
| 6q | 3 (3,8%) | 1 (2,4%) | 91 (23%) | *** |
| 7p | 8 (10%) | 15 (37%) | 283 (70%) | *** |
| 7q | 7 (9,0%) | 15 (37%) | 247 (62%) | *** |
| 8p | 19 (24%) | 7 (17%) | 106 (26%) | NS |
| 8q | 24 (30%) | 12 (29%) | 226 (56%) | ** |
| 9p | 6 (7,7%) | 8 (20%) | 89 (22%) | * |
| 9q | 6 (7,7%) | 8 (20%) | 71 (18%) | NS |
| 10p | 1 (1,2%) | 0 | 45 (11%) | ** |
| 10q | 1 (1,2%) | 0 | 26 (6,5%) | NS |
| 11p | 0 | 0 | 72 (18%) | ** |
| 11q | 0 | 0 | 66 (16,4%) | ** |
| 12p | 16 (20%) | 9 (22%) | 82 (20%) | NS |
| 12q | 16 (20%) | 9 (22%) | 69 (17%) | NS |
| 13q | 11 (14%) | 3 (7,3%) | 299 (75%) | *** |
| 14q | 2 (2,5%) | 1 (2,4%) | 36 (9,0%) | NS |
| 15q | 0 | 0 | 17 (4,2%) | NS |
| 16p | 1 (1,2%) | 0 | 126 (31%) | *** |
| 16q | 1 (1,2%) | 0 | 124 (31%) | *** |
| 17p | 3 (3,8%) | 0 | 23 (5,7%) | NS |
| 17q | 3 (3,8%) | 1 (2,4%) | 80 (5,7%) | NS |
| 18p | 7 (9,0%) | 2 (4,8%) | 18 (4,5%) | NS |
| 18q | 7 (9,0%) | 2 (4,8%) | 8 (2,0%) | NS |
| 19p | 2 (2,5%) | 3 (7,3%) | 81 (20%) | NS |
| 19q | 3 (3,8%) | 7 (17%) | 83 (20%) | ** |
| 20p | 12 (15%) | 5 (12%) | 271 (68%) | *** |
| 20q | 13 (17%) | 8 (20%) | 371 (93%) | *** |
| 21q | 0 | 2 (4,8%) | 31 (7,7%) | NS |
| 22q | 1 (1,2%) | 0 | 16 (4,0%) | NS |

Supplementary table 2. Deletion frequency of chromosome arms in HM, HM-like and non-HM tumors.

| Chromosome arm | HM | HM_like | non_HM | P-value |
| --- | --- | --- | --- | --- |
| 1p | 2 (2,5%) | 0 | 117 (29%) | *** |
| 1q | 2 (2,5%) | 0 | 59 (14 %) | *** |
| 2p | 0 | 0 | 10 (2,5%) | NS |
| 2q | 0 | 0 | 10 (2,5%) | NS |
| 3p | 2 (2,5%) | 2 (4,8%) | 52 (13%) | ** |
| 3q | 2 (2,5%) | 1 (2,4%) | 34 (8,5%) | NS |
| 4p | 2 (2,5%) | 2 (4,8%) | 165 (41%) | *** |
| 4q | 2 (2,5%) | 2 (4,8%) | 160 (39%) | *** |
| 5p | 2 (2,5%) | 1 (2,4%) | 59 (15%) | *** |
| 5q | 3 (3,8%) | 2 (4,8%) | 97 (24%) | *** |
| 6p | 3 (3,8%) | 0 | 39 (10%) | NS |
| 6q | 4 (5,1%) | 0 | 51 (13%) | ** |
| 7p | 0 | 0 | 5 (1,2%) | NS |
| 7q | 0 | 0 | 7 (1,7%) | NS |
| 8p | 2 (2,5%) | 7 (17%) | 159 (40%) | *** |
| 8q | 0 | 1 (2,4%) | 24 (6%) | NS |
| 9p | 0 | 0 | 66 (16%) | *** |
| 9q | 0 | 0 | 65 (16%) | *** |
| 10p | 1 (1,2%) | 3 (7,3%) | 82 (20%) | *** |
| 10q | 1 (1,2%) | 2 (4,8%) | 87 (22%) | *** |
| 11p | 1 (1,2%) | 3 (7,3%) | 61 (15%) | *** |
| 11q | 0 | 4 (9,7%) | 70 (17%) | *** |
| 12p | 0 | 0 | 55 (14%) | ** |
| 12q | 0 | 1 (2,4%) | 55 (14%) | ** |
| 13q | 0 | 1 (2,4%) | 12 (3%) | NS |
| 14q | 1 (1,2%) | 0 | 165 (41%) | *** |
| 15q | 1 (1,2%) | 4 (9,7%) | 185 (46%) | *** |
| 16p | 3 (3,8%) | 2 (4,8%) | 25 (6,2%) | NS |
| 16q | 2 (2,5%) | 3 (7,3%) | 30 (7,5%) | NS |
| 17p | 4 (5,1%) | 5 (12%) | 262 (65%) | *** |
| 17q | 3 (3,8%) | 2 (4,8%) | 73 (18%) | *** |
| 18p | 0 | 3 (7,3%) | 315 (79%) | *** |
| 18q | 2 (2,5%) | 5 (12%) | 337 (84%) | *** |
| 19p | 1 (1,2%) | 1 (2,4%) | 52 (13%) | ** |
| 19q | 1 (1,2%) | 1 (2,4%) | 47 (11%) | * |
| 20p | 2 (2,5%) | 0 | 54 (13%) | ** |
| 20q | 0 | 0 | 2 | NS |
| 21q | 8 (10%) | 4 (9,7%) | 147 (37%) | *** |
| 22q | 2 (2,5%) | 2 (4,8%) | 161 (40%) | *** |

*Supplementary table 5. Genes Belonged to interferon pathway that had higher expression in HM-like patients compared to non-HM tumors. This is essentially a numeric matrix with elements -1, 0 or 1 depending on whether each t-statistic is classified as significantly negative, not significant, or significantly positive*

|  | HM-like vs non-HM | HM vs non-HM | HM vs HMlike | LFC Hm vs non-hm | LFC Hm-like vs non-Hm | LFC Hm vs Hm-like |
| --- | --- | --- | --- | --- | --- | --- |
| APOL1 | 1 | 1 | 0 | 1.347450445 | 0.84466823 | 0 |
| APOL6 | 1 | 1 | 1 | 0.940863518 | 0.431600442 | 0.509263076 |
| DDX60 | 1 | 1 | 0 | 0.861606689 | 0.458314365 | 0 |
| IFI16 | 1 | 1 | 0 | 0.81516748 | 0.641880483 | 0 |
| OASL | 1 | 1 | 0 | 1.085462258 | 0.633036489 | 0 |
| PML | 1 | 1 | 0 | 0.625011075 | 0.372482984 | 0 |
| RTP4 | 1 | 1 | 0 | 0.725814106 | 0.439016435 | 0 |
| SAMD9 | 1 | 1 | 0 | 1.05357992 | 0.602912022 | 0 |
| SAMD9L | 1 | 1 | 1 | 1.469221765 | 0.801797748 | 0.667424016 |
| TRIM22 | 1 | 1 | 0 | 0.937724279 | 0.717083853 | 0 |
| TRIM5 | 1 | 1 | 0 | 0.283549438 | 0.188214593 | 0 |
| USP18 | 1 | 1 | 1 | 1.427404003 | 0.598758404 | 0.828645599 |


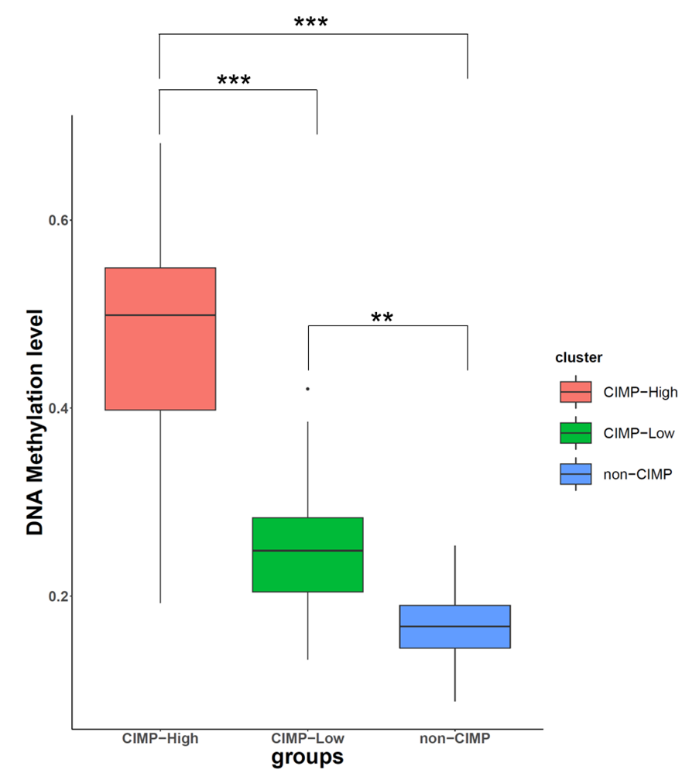

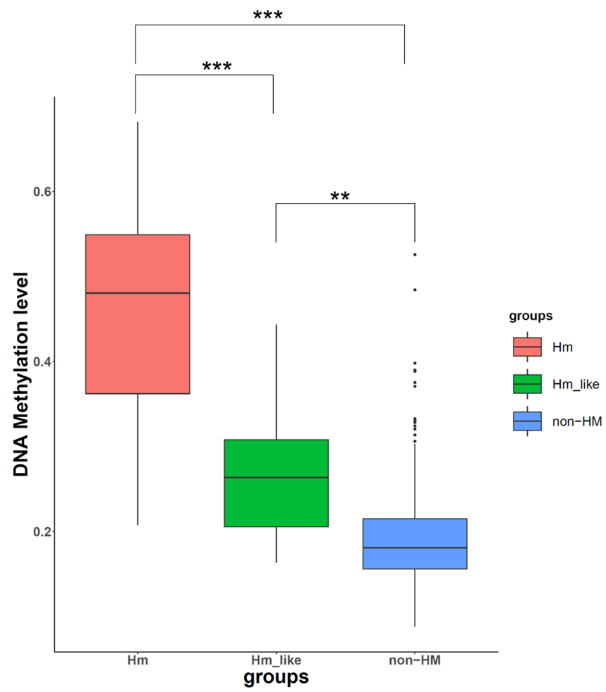
**Supplementary figures**

Supplementary figure 1. DNA methylation levels of the 1000 most differentially methylated probes across the three clusters. The boxplots show DNA methylation level across the clusters identified in the unsupervised hierarchical analysis. Statistical analysis of data was performed using t-test : ***p < .001, **p < .01.

Supplementary figure 2. DNA methylation levels of the 1000 most differentially methylated probes across the HM, HM-like and non-HM subgroups. The boxplots show DNA methylation level across the three subgroups. Statistical analysis of data was performed using t-test : ***p < .001, **p < .01.


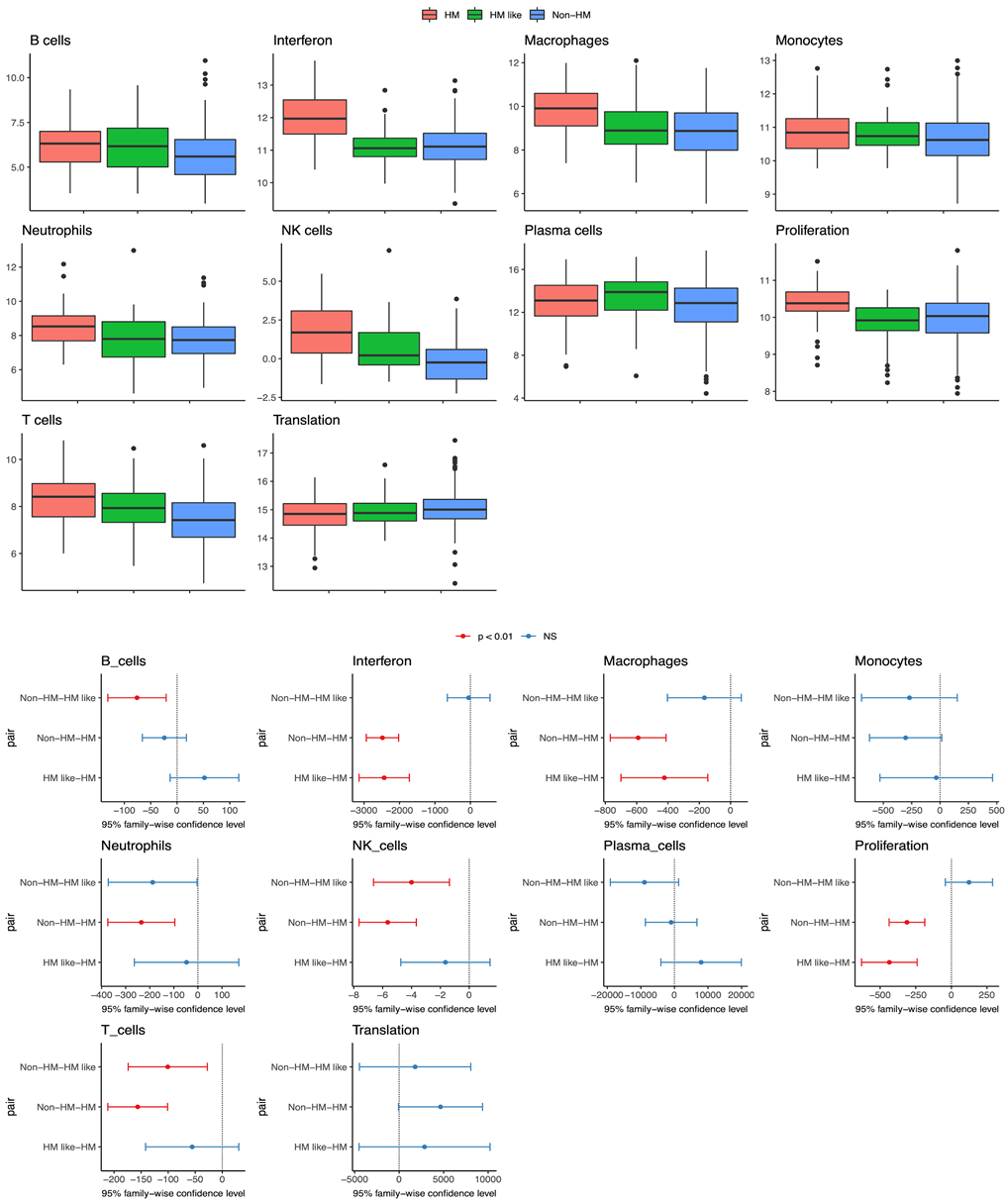


Supplementary figure 3. Results of immune signatures analysis performed by ImSig. The Boxplots shows the gene expression of immune signatures genes (estimated relative abundance) across the HM, non-HM and HM-like groups. The Tuckey plots show the comparison of the mean expression (i.e., difference of the mean) of the immune signatures between the three groups. A 95% confidence level of the difference of the means is reported. Lines are coloured according to p-value resulting from multiple comparison Tuckey test: red (p-value <0.01), blue (p-value > non-significant).
